# Graft and Patient Survival in Kidney Transplant Recipients Over the Age of Sıxty-Five

**DOI:** 10.7759/cureus.20913

**Published:** 2022-01-03

**Authors:** Gulay Yilmaz, Ebru Ozdemir, Murat Yildar, Hamit Karayagiz, Ibrahim Berber, Ulkem Cakir

**Affiliations:** 1 Department of Nephrology and Transplantation, Acibadem International Hospital, Istanbul, TUR; 2 Organ Transplantation Center, Acibadem International Hospital, Istanbul, TUR; 3 General Surgery, Istanbul Esenyurt Necmi Kadioglu Public Hospital, Istanbul, TUR; 4 Organ Transplantation Center, Acibadem University, Istanbul, TUR; 5 Nephrology, Acibadem University, Istanbul, TUR

**Keywords:** chronic kidney disease (ckd), mortality, kidney transplantation, graft survey, geriatrics

## Abstract

Introduction: Elderly patients have increased morbidity and mortality compared to younger patients due to existing comorbid diseases and chronic immunosuppression. Therefore, the option of kidney transplantation for renal replacement therapy in elderly patients is still being controversial. Our aim in this study was to evaluate graft function, graft and patient survival, and associated factors in kidney transplant recipients over 65 years of age, at 11 years of follow-up.

Methods: The study included 53 patients aged 65-76 years, out of a total of 1319 patients who underwent live kidney transplantation in the Organ Transplant Center of Acibadem International Hospital between October 2010 and July 2021. Demographic characteristics and creatinine values were recorded. Graft survival rates and patient survival rates at one, three, and five years were analyzed.

Results: Fifty-three patients, 14 female, 39 male, aged 65-76 years were included in the study. The follow-up period of the patients was 7-125 months. During the follow-up, 20 patients died. Graft loss occurred in two of 20 patients who died, and 18 patients died with working grafts. Graft loss developed in two of the 33 surviving patients. In the whole group, one-, three-, and five-year patient survival rates were 94%, 81%, and 76%, respectively.

Conclusion: These results emphasize that kidney transplantation is a viable treatment option in elderly patients who have been well evaluated before kidney transplantation.

## Introduction

In recent years, the number of kidney transplant patients over the age of 65 has increased significantly due to both the increase in the number of patients with chronic kidney disease and the prolongation of the life expectancy in these patients [1]. The best renal replacement therapy for elderly patients has been frequently debated.

When elderly kidney transplant patients and elderly dialysis patients are compared, it is seen that the quality of life and life expectancy are better in kidney transplant patients [2]. Actually, this is true for all age groups with chronic kidney disease (CKD). Patients over the age of 65 are at greater risk for comorbidities, immunosuppression-related complications, and chronic graft failure more than younger patients. It is also difficult to make a decision in favor of operation for these patients who have many comorbidities, decreased functional status, frailty, and cognitive impairment [3]. But we also know that older hemodialysis patients have a high prevalence of depression, lower quality of life, and shorter lifetime than older transplant patients [4,5]. In a study from the United States, it was shown that elderly adults undergoing home hemodialysis had a risk of mortality that was five times as high as that of kidney transplant recipients [6]. Wolfe et al. compared survival in two groups. These two groups were consisted of hemodialysis patients and kidney transplant patients. Survival advantage was shown in kidney transplant patients across all ages when compared to staying on dialysis [7]. Age-related comorbidities like ischemic heart disease, frailty and the other vascular diseases are risk factors for postoperative complications and death. Our knowledge about elderly patients’ outcomes after transplantation is still not enough. So, we also wanted to evaluate graft function, graft and patient survival, and associated factors in kidney transplant recipients over 65 years of age.

## Materials and methods

The study included 59 patients aged 65-76 years, out of a total of 1319 patients who underwent live kidney transplantation in Acibadem International Hospital Organ Transplantation Center between October 2010 and July 2021. Six patients who did not come to the outpatient follow-up regularly and whose final kidney function tests were unknown were excluded from the study. This study was approved by the Ethical Committee of Acibadem University in October 2021 with the approval number 202-20/13.

Study design

The files of 53 patients were reviewed retrospectively. Donors’ age and gender, recipients’ age, gender, etiology of chronic kidney disease, time after transplant, type of renal replacement therapy, human leukocyte antigen mismatch counts, panel reactive antibody status, and creatinine values were recorded. All of the patients had low immunological risk, but all of them received induction therapy with 2-2.5 mg/kg anti-T-lymphocyte globulin according to our immunosuppressive therapy protocol. They received 1000 mg steroid for the first day, 500 mg second day, 250 mg third day, 180 mg fourth day, and then 20 mg. We tapered the dose every 15 days. After two months they received 5 mg. Baseline immunosuppression of all patients included calcineurin inhibitor, antimetabolite (mycophenolic acid or mycophenolate mofetil), and steroids for all patients. All patients were followed up weekly in the first month after the transplant, every 15 days in the next two months, once a month in the first year and then they were followed up every three months. The information about patients who died outside the hospital was learned by entering our country's organ transplant data system. Graft survival and patient survival rates at one, three, and five years were calculated. 

Statistical analysis

The results were analyzed by use of the Statistical Package for the Social Sciences, version 22.0 ( IBM Corp., Armonk, NY). Values displaying a normal distribution are expressed as mean ± standard deviation (SD). Differences between numeric variables were tested with the use of the independent-samples Student t-test or Mann-Whitney U test, whichever was appropriate. Ratios for categorical variables were compared by use of Chi-square tests. A value was considered statistically significant at p <0.05.

## Results

Fifty-three patients, 14 female, 39 male, aged 65-76 years were included in the study. The mean recipient age was 67.2 (±2.7) and 4% of our transplant recipients (n=1359) during 11 years of follow-up were over the age of 65. The mean donor age was 53.1 (±12.1) years and 32 (60.4%) of donors were female. Etiology of end-stage renal disease was; diabetes mellitus (28.3%, n=15), hypertension (28.3%, n=15), glomerulonephritis (5.7%, n=3), tubulointerstitial nephritis (13.2%, n=7), polycystic kidney disease (5.7%, n=3) and the other’s etiology were unknown. We analyzed comorbidities and the survival rate, and only hypertension was found to be associated with patient survival (p=0,029 r=0,835).

The follow-up period of the patients was 7-125 (mean:63.5) months. During the follow-up, 20 patients died. Graft loss occurred in two of 20 patients who died while 18 patients died with functioning grafts. The causes of death were cardiovascular causes in eight patients, infection in eight patients, malignancy in two patients, and cerebrovascular causes in two patients.

There was no difference between the survival and non-survival groups in terms of human leukocyte antigen (HLA) mismatch (MM) count (4.4±1.3 and 4.5±1.4 respectively, p=0.9). Panel reactive antibody (PRA) Class 1 was positive in five (25%) of the 20 patients who died. PRA Class 2 was also positive in five (25%) of the 20 patients who died. PRA Class 1 was positive in seven (21%) of the 33 patients who were alive and PRA Class 2 was also positive in seven (21%) of the 33 patients who were alive. When we analyzed all groups, PRA Class 1 was positive in 12 patients and PRA Class 2 was positive in 12 patients. Both PRA Class 1 and Class 2 were positive in six patients (Table 1). While the HLA-MM count of four patients with graft loss (n=4) was 3.2±1.5, the HLA-MM count of patients without graft loss (n=49) was 4.5±1.2 (p=0.05). Graft loss developed in two of the 33 (6%) surviving patients and in two of the non-survivors. Death censored graft survival rates were 96%, 92%, and 92% for one-, three-, and five-year respectively. In the whole group, one-, three-, and five-year patient survival rates were 94%, 81%, and 76%, respectively (Table 1). The last creatinine level of the patients who died was 1.9±0.2 mg/dl and surviving patients’ creatinine level was 1.6±0.2 mg/dl.

**Table 1:** Demographic and clinical findings of patients CKD, chronic kidney disease; DM, diabetes mellitus; GN, glomerulonephritis; HLA, human leukocyte antigen; HT, hypertension; MM, mismatch; PCK, polycystic kidney disease; PRA, panel reactive antibody; TIN, tubulointerstitial nephritis

Main baseline characteristics	All Patients n(%)
Recipient Gender; n(%)	
Female	14 (26.4)
Male	39 (73.6)
Recipient Age (year)	67.2 (±2.7)
Donor Gender; n(%)	
Female	32 (60.4)
Male	21 (39.6)
Donor Age (year)	53.1 (±12.1)
Etiology of CKD; n(%)	
DM	15 (28.3)
HT	15(28.3)
TIN	7 (13.2)
GN	3 (5.7)
PCK	3 (5.7)
OTHERS	9 (16.9)
Renal Replacement Therapy; n(%)	
Preemptive	15 (28.3)
Dialysis	38 (71.7)
Total HLA-MM Count	
0-2 MM	5 (9.4)
2-4 MM	17 (32)
4-6 MM	31 (58.4)
Pretransplant PRA positivity; n(%)	
Class 1 (+)	12 (22)
Class 2 (+)	12 (22)
Class 1 and Class 2 (+)	6(8.8)

## Discussion

The aim of this study was to evaluate the graft and patient survival, and associated factors in kidney transplant recipients over 65 years of age. As an overall interpretation of our findings, 4% of our transplant recipients during 11 years of follow-up were over the age of 65 and the five-year patient survival rate was 76%.

The number of patients over the age of 65 is increasing day by day. There is a transition from a young population structure to an aging population structure throughout the world. So the elderly patient population is also increasing rapidly. The number of elderly end-stage renal disease (ESRD) patients is also increasing. As stated above 4% of our transplant recipients during 11 years of follow-up were over the age of 65. According to the 2019 registry report of the Turkish Society of Nephrology, in the last ten years, 3.3% of patients who had kidney transplantation in our country were over the age of 65 [8]. It is possible to say that our patient group is slightly older than the Turkish average. The choice of renal replacement therapy for elderly patients is probably the most difficult question for nephrologists. Elderly ESRD patients have many comorbidities and social, physical, psychological challenges [9]. These challenges like frailty, falls, and functional impairment increase the risk of mortality in CKD patients [10,11]. When choosing the modality of renal replacement therapy life expectancy also becomes an important parameter for clinicians. The other parameter which affects the choice of renal replacement therapy is the life quality of older patients. The quality of life of older patients is lower than younger patients. Kidney transplantation provides older patients with a longer lifespan and a more comfortable life. In a study from Iran, they compared the quality of life of renal-transplanted and hemodialysis patients and they revealed that the quality-of-life mean scores of renal transplantation patients were better than hemodialysis patients (21.36 [SD, 4.06] vs 20.35 [SD, 5.14]; p = 0.03) [12]. Zhang et al. also analyzed the factors affecting the quality of life in CKD patients, and they showed that age was one of the main factors affecting the quality of life in CKD patients. They also showed that kidney transplantation is superior to hemodialysis and peritoneal dialysis in terms of quality of life [13]. According to the United States renal data system (USRDS) Annual Data Report, the survival of elderly dialysis patients is poor. In USRDS Annual Data Report 2016, it was revealed that in the United States, mortality of patients who are on dialysis and aged 65 to 74 years is twice that of younger patients; and patients over age 75 who are on dialysis have a threefold increased risk of mortality compared to younger patients on dialysis [14,15]. When compared to younger recipients, older recipients have a lower patient and graft survival [16]. But when we compare the survival of hemodialysis and transplant patients, transplant patients have better survival than hemodialysis patients. For older patients who are on a waiting list for a transplant, the projected lifespan is 6 years, but it is 10 years for patients who have kidney transplants [7]. In a study from Scotland 325 patients, >60 years old who were on the list of kidney transplantation were followed. During the follow-up 128 (39.4%) received a transplant and the other 197 (60.6%) continued undergoing dialysis. Transplant recipients had less ischemic heart disease (p = 0.024) and cerebrovascular disease (p = 0.03). The overall mortality rate was lower in the transplant group. There was a significantly lower risk of death for transplant group (relative risk [RR] = 0.35, 95% CI 0.22-−0.54; p<0.0001, log-rank) [17].

The mortality rate of our patients during the follow-up (63.5 months) was 37%. This was similar to other studies. Karim et al. showed that the mortality rate was 31.9% for recipients aged 70-79 years and 22% for the others aged 60-69 years after a median follow-up of 4.4 years [18]. Our follow-up duration was 7-125 (mean:63.5) months. During the follow-up 20 (37%) of 53 patients died. The main causes of death were cardiovascular diseases and infections. In Karim et al's study, the main causes of death were cardiovascular diseases (21%), infection (21%), and malignancy (20%), which was similar to our results [18]. For our patients, one-, three-, and five-year patient survival rates were 94%, 81%, 76%, and graft survival rates were 96%, 92%, and 92% respectively. These were also consistent with the literature. According to the study of Adani et al., at one-, three-, five-, and 10-years, overall patient survival was 89%, 84%, 72%, and 45%, and overall graft survival was 100%, 97%, 89%, and 84%, respectively. In this study causes of death were infections in 42%, tumors in 23%, cardiovascular disease in 14%, cerebrovascular disease in 7%, and unknown in 14% [19]. When we analyzed the graft survey, we saw that graft loss developed in two of the 33 (6%) surviving patients and 18 patients died with functioning grafts. We think that this is related to the higher mortality rate with surviving grafts in older transplant recipients than in younger ones. On the contrary, Lufft et al. compared two groups of kidney transplants. The first group was over the age of 65 and the other group was younger. During the follow-up of 5 years, acute rejections were more frequent in the older transplant patients, but graft losses due to rejections were not more frequent in younger patients than in older ones [20].

We compared the HLA-MM counts, pretransplant PRA Class 1 and Class 2 positivity of survival and non-survival patients and we saw that there was no difference between the groups. Contrary to our findings Lim et al. reported that patients with higher PRA levels were found to have higher mortality rates [21]. But their results were independent of age. Arman et al. also evaluated mortality, graft failure, and predictors of these outcomes in elderly transplant patients. They reported that chronic heart failure, PRA >10%, delayed graft function, and acute cellular rejection were risk factors for graft failure, but for mortality, only chronic heart failure and chronic heart disease were risk factors [22]. We think that this may be related to the lower immunological risk in elderly patients compared to younger ones. Younger patients, African Americans, higher HLA-mismatch counts, PRA positivity, pretransplant donor-specific antibody positivity, pretransplant blood transfusion, and previous pregnancies increase the immunological risk of the patient. In our study, the HLA-MM count of patients without graft loss (n=49) was higher than the patients with graft loss (n=4). We think that this may be related to the low number of patients with graft loss.

## Conclusions

Our study emphasizes that, although elderly patients with chronic kidney disease have more comorbidities, kidney transplantation is a viable treatment option for these patients. The main point about elderly patients is that these patients should be evaluated in great detail before the operation, and after the operation, they should be monitored very closely for complications and drug side effects.
